# Supplementary material for: Modeling glioblastoma heterogeneity as a dynamic network of cell states
Source: Mol Syst Biol. 2021 Sep 16;17(9):e10105. doi: 10.15252/msb.202010105 (PMC8444284; doi:10.15252/msb.202010105)
Supplement: Supplementary file 6 — Source Data for Figure 5 [file MSB-17-e10105-s004.zip › Figure5A_sourcedata/GSEA_3017/nosigdb_stateC.GseaPreranked.1622795002785/RICHARDS_NOWAKOWSKI_SCIENCE_2017_MGE_DIV_UPREG.html]

Details for gene set RICHARDS\_NOWAKOWSKI\_SCIENCE\_2017\_MGE\_DIV\_UPREG[GSEA]

|  || Dataset | state13017 |
| Phenotype | NoPhenotypeAvailable |
| Upregulated in class | na\_pos |
| GeneSet | RICHARDS\_NOWAKOWSKI\_SCIENCE\_2017\_MGE\_DIV\_UPREG |
| Enrichment Score (ES) | 0.7985125 |
| Normalized Enrichment Score (NES) | 7.0049257 |
| Nominal p-value | 0.0 |
| FDR q-value | 0.0 |
| FWER p-Value | 0.0 |
Table: GSEA Results Summary

  

Fig 1: Enrichment plot: RICHARDS\_NOWAKOWSKI\_SCIENCE\_2017\_MGE\_DIV\_UPREG      
 Profile of the Running ES Score & Positions of GeneSet Members on the Rank Ordered List

  

| PROBE | GENE SYMBOL | GENE\_TITLE | RANK IN GENE LIST | RANK METRIC SCORE | RUNNING ES | CORE ENRICHMENT || 1 | SYNE2 |  |  | 0 | 0.839 | 0.0177 | Yes |
| 2 | TFDP2 |  |  | 1 | 0.787 | 0.0343 | Yes |
| 3 | RND3 |  |  | 2 | 0.713 | 0.0493 | Yes |
| 4 | HES6 |  |  | 5 | 0.694 | 0.0597 | Yes |
| 5 | MAGI1 |  |  | 6 | 0.652 | 0.0735 | Yes |
| 6 | ATAD2 |  |  | 7 | 0.649 | 0.0872 | Yes |
| 7 | FAM111B |  |  | 8 | 0.616 | 0.1002 | Yes |
| 8 | HIST1H4C |  |  | 9 | 0.603 | 0.1129 | Yes |
| 9 | CENPU |  |  | 10 | 0.588 | 0.1253 | Yes |
| 10 | HIST1H1C |  |  | 11 | 0.583 | 0.1376 | Yes |
| 11 | HIST1H1D |  |  | 12 | 0.583 | 0.1499 | Yes |
| 12 | FAM111A |  |  | 13 | 0.576 | 0.1620 | Yes |
| 13 | EZH2 |  |  | 14 | 0.574 | 0.1741 | Yes |
| 14 | CCNE2 |  |  | 15 | 0.570 | 0.1861 | Yes |
| 15 | CLSPN |  |  | 17 | 0.561 | 0.1959 | Yes |
| 16 | C21orf58 |  |  | 18 | 0.559 | 0.2076 | Yes |
| 17 | RRM2 |  |  | 19 | 0.542 | 0.2191 | Yes |
| 18 | HMGB2 |  |  | 20 | 0.534 | 0.2303 | Yes |
| 19 | CDK6 |  |  | 24 | 0.518 | 0.2349 | Yes |
| 20 | XRCC2 |  |  | 26 | 0.495 | 0.2433 | Yes |
| 21 | CHAF1A |  |  | 27 | 0.494 | 0.2537 | Yes |
| 22 | UBE2T |  |  | 31 | 0.475 | 0.2574 | Yes |
| 23 | DLX1 |  |  | 32 | 0.469 | 0.2672 | Yes |
| 24 | HIST1H1B |  |  | 35 | 0.460 | 0.2727 | Yes |
| 25 | CDCA7L |  |  | 37 | 0.453 | 0.2802 | Yes |
| 26 | GMNN |  |  | 38 | 0.453 | 0.2897 | Yes |
| 27 | ORC6 |  |  | 39 | 0.450 | 0.2992 | Yes |
| 28 | MMS22L |  |  | 40 | 0.448 | 0.3087 | Yes |
| 29 | ESCO2 |  |  | 43 | 0.440 | 0.3137 | Yes |
| 30 | UHRF1 |  |  | 45 | 0.436 | 0.3208 | Yes |
| 31 | RAD51AP1 |  |  | 46 | 0.435 | 0.3300 | Yes |
| 32 | PCNA |  |  | 48 | 0.435 | 0.3371 | Yes |
| 33 | TMPO |  |  | 49 | 0.432 | 0.3462 | Yes |
| 34 | DTL |  |  | 50 | 0.430 | 0.3552 | Yes |
| 35 | HIST1H1E |  |  | 51 | 0.430 | 0.3643 | Yes |
| 36 | TOP2A |  |  | 52 | 0.426 | 0.3733 | Yes |
| 37 | SPC25 |  |  | 53 | 0.415 | 0.3820 | Yes |
| 38 | BRCA1 |  |  | 54 | 0.410 | 0.3907 | Yes |
| 39 | DHFR |  |  | 55 | 0.405 | 0.3992 | Yes |
| 40 | WDR76 |  |  | 56 | 0.403 | 0.4077 | Yes |
| 41 | TYMS |  |  | 60 | 0.390 | 0.4096 | Yes |
| 42 | RRM1 |  |  | 62 | 0.386 | 0.4157 | Yes |
| 43 | SMC2 |  |  | 63 | 0.386 | 0.4238 | Yes |
| 44 | RAB3IP |  |  | 66 | 0.382 | 0.4276 | Yes |
| 45 | GLCCI1 |  |  | 68 | 0.378 | 0.4335 | Yes |
| 46 | E2F7 |  |  | 69 | 0.377 | 0.4414 | Yes |
| 47 | PSIP1 |  |  | 73 | 0.374 | 0.4430 | Yes |
| 48 | DEK |  |  | 74 | 0.373 | 0.4509 | Yes |
| 49 | NASP |  |  | 75 | 0.371 | 0.4587 | Yes |
| 50 | POLD3 |  |  | 76 | 0.367 | 0.4665 | Yes |
| 51 | SMC4 |  |  | 78 | 0.365 | 0.4721 | Yes |
| 52 | ITGB3BP |  |  | 79 | 0.364 | 0.4797 | Yes |
| 53 | CDCA5 |  |  | 81 | 0.361 | 0.4852 | Yes |
| 54 | GINS2 |  |  | 83 | 0.360 | 0.4907 | Yes |
| 55 | STMN1 |  |  | 85 | 0.357 | 0.4961 | Yes |
| 56 | BARD1 |  |  | 86 | 0.357 | 0.5036 | Yes |
| 57 | DUT |  |  | 87 | 0.356 | 0.5112 | Yes |
| 58 | MIS18BP1 |  |  | 89 | 0.355 | 0.5165 | Yes |
| 59 | MCM7 |  |  | 91 | 0.352 | 0.5218 | Yes |
| 60 | USP1 |  |  | 92 | 0.352 | 0.5293 | Yes |
| 61 | ATAD5 |  |  | 97 | 0.345 | 0.5281 | Yes |
| 62 | FEN1 |  |  | 98 | 0.342 | 0.5353 | Yes |
| 63 | NUSAP1 |  |  | 100 | 0.340 | 0.5404 | Yes |
| 64 | H1FX |  |  | 101 | 0.339 | 0.5475 | Yes |
| 65 | CKB |  |  | 102 | 0.335 | 0.5546 | Yes |
| 66 | NCAPD3 |  |  | 103 | 0.334 | 0.5616 | Yes |
| 67 | MCM8 |  |  | 104 | 0.331 | 0.5686 | Yes |
| 68 | SLBP |  |  | 106 | 0.331 | 0.5735 | Yes |
| 69 | ZNF43 |  |  | 107 | 0.330 | 0.5804 | Yes |
| 70 | BEST3 |  |  | 110 | 0.327 | 0.5831 | Yes |
| 71 | MCM3 |  |  | 114 | 0.324 | 0.5836 | Yes |
| 72 | H2AFX |  |  | 115 | 0.322 | 0.5904 | Yes |
| 73 | HIRIP3 |  |  | 116 | 0.321 | 0.5972 | Yes |
| 74 | TIMELESS |  |  | 119 | 0.319 | 0.5997 | Yes |
| 75 | CCDC34 |  |  | 120 | 0.318 | 0.6064 | Yes |
| 76 | HAT1 |  |  | 121 | 0.318 | 0.6131 | Yes |
| 77 | FBXW7 |  |  | 122 | 0.316 | 0.6197 | Yes |
| 78 | RHOBTB3 |  |  | 124 | 0.313 | 0.6242 | Yes |
| 79 | SMC1A |  |  | 125 | 0.312 | 0.6308 | Yes |
| 80 | HMGN2 |  |  | 126 | 0.312 | 0.6374 | Yes |
| 81 | DNMT1 |  |  | 127 | 0.311 | 0.6440 | Yes |
| 82 | STAG2 |  |  | 128 | 0.309 | 0.6505 | Yes |
| 83 | FBXO5 |  |  | 130 | 0.308 | 0.6549 | Yes |
| 84 | H3F3A |  |  | 132 | 0.304 | 0.6592 | Yes |
| 85 | KIF15 |  |  | 136 | 0.302 | 0.6592 | Yes |
| 86 | NCAPG |  |  | 137 | 0.301 | 0.6655 | Yes |
| 87 | RFC4 |  |  | 138 | 0.300 | 0.6719 | Yes |
| 88 | CENPK |  |  | 139 | 0.299 | 0.6782 | Yes |
| 89 | LMNB1 |  |  | 142 | 0.293 | 0.6801 | Yes |
| 90 | ASF1B |  |  | 143 | 0.291 | 0.6863 | Yes |
| 91 | ASCL1 |  |  | 145 | 0.290 | 0.6903 | Yes |
| 92 | C12orf57 |  |  | 146 | 0.289 | 0.6964 | Yes |
| 93 | CCND2 |  |  | 149 | 0.288 | 0.6982 | Yes |
| 94 | SIVA1 |  |  | 151 | 0.286 | 0.7021 | Yes |
| 95 | SUPT16H |  |  | 153 | 0.286 | 0.7061 | Yes |
| 96 | RFC3 |  |  | 156 | 0.283 | 0.7078 | Yes |
| 97 | RNASEH2B |  |  | 158 | 0.282 | 0.7117 | Yes |
| 98 | ZNF93 |  |  | 160 | 0.281 | 0.7155 | Yes |
| 99 | PARP1 |  |  | 163 | 0.280 | 0.7172 | Yes |
| 100 | MYBL2 |  |  | 164 | 0.278 | 0.7230 | Yes |
| 101 | H2AFY |  |  | 165 | 0.278 | 0.7289 | Yes |
| 102 | CHD7 |  |  | 166 | 0.277 | 0.7347 | Yes |
| 103 | VRK1 |  |  | 169 | 0.275 | 0.7363 | Yes |
| 104 | HELLS |  |  | 171 | 0.271 | 0.7399 | Yes |
| 105 | C19orf48 |  |  | 173 | 0.271 | 0.7435 | Yes |
| 106 | MAD2L1 |  |  | 174 | 0.270 | 0.7492 | Yes |
| 107 | RBBP8 |  |  | 177 | 0.267 | 0.7506 | Yes |
| 108 | RIF1 |  |  | 178 | 0.266 | 0.7562 | Yes |
| 109 | POLA1 |  |  | 179 | 0.266 | 0.7618 | Yes |
| 110 | MSH2 |  |  | 180 | 0.265 | 0.7674 | Yes |
| 111 | DDX11 |  |  | 181 | 0.265 | 0.7730 | Yes |
| 112 | RMI1 |  |  | 183 | 0.263 | 0.7764 | Yes |
| 113 | NAP1L4 |  |  | 184 | 0.262 | 0.7819 | Yes |
| 114 | FANCL |  |  | 185 | 0.261 | 0.7874 | Yes |
| 115 | NCAPG2 |  |  | 187 | 0.260 | 0.7908 | Yes |
| 116 | MCM4 |  |  | 189 | 0.259 | 0.7942 | Yes |
| 117 | CDK1 |  |  | 191 | 0.255 | 0.7974 | Yes |
| 118 | E2F3 |  |  | 194 | 0.252 | 0.7985 | Yes |
| 119 | CLIC1 |  |  | 237 | -0.261 | 0.7154 | No |
| 120 | HSPA5 |  |  | 447 | -0.350 | 0.2819 | No |
| 121 | RAB13 |  |  | 482 | -0.388 | 0.2183 | No |
| 122 | LDHA |  |  | 498 | -0.412 | 0.1954 | No |
| 123 | DBI |  |  | 514 | -0.440 | 0.1730 | No |
Table: GSEA details [plain text format]

  

Fig 2: RICHARDS\_NOWAKOWSKI\_SCIENCE\_2017\_MGE\_DIV\_UPREG: Random ES distribution      
 Gene set null distribution of ES for **RICHARDS\_NOWAKOWSKI\_SCIENCE\_2017\_MGE\_DIV\_UPREG**
